# Evaluation of pregnancy outcomes using a novel hysterosalpingography scoring system for tubal patency

**DOI:** 10.1016/j.eurox.2025.100437

**Published:** 2025-11-27

**Authors:** Huijun Yang, Yali Xu, Saiming Cheng, Haixia Zhang, Jiejun Cheng, Feng Gao

**Affiliations:** a Department of radiology, Shanghai First Maternity and Infant Hospital, Tongji University School of Medicine, Shanghai 201204, China; b Department of radiology, Shanghai tenth people’s Hospital, Tongji University School of Medicine, Shanghai 201072, China

**Keywords:** Hysterosalpingography, Fertility, Pregnancy rate, Tube patency, Hydrosalpinx

## Abstract

**Objective:**

This study aims to evaluate pregnancy rates using a novel classification system based on hysterosalpingography (HSG) findings, categorizing tubal patency as either patent or functionally deficient. Furthermore, the study assesses the predictive value of this scoring system for fertility outcomes.

**Methods:**

A retrospective cohort study was conducted involving 4461 patients who underwent HSG at an academic radiology center between January 2020 and December 2022. HSG findings and subsequent pregnancy outcomes were systematically analyzed.

**Results:**

Of the 2461 patients initially followed up, 301 were excluded due to incomplete data, resulting in a final cohort of 2160 patients. Within two years post-HSG, 970 patients (44.9 %) achieved natural pregnancy, 808 (83.2 %) of whom resulted in live births. In vitro fertilization (IVF) pregnancies accounted for 35.8 % (n = 775). Ectopic pregnancies occurred in 1.8 % of patients, with a higher incidence in those with unilateral tubal occlusion. Using the novel scoring system—where Score 0 indicates bilateral patency, Score 1 indicates unilateral functional deficiency, and Score 2 indicates bilateral functional deficiency—the Score 0 group exhibited the highest natural pregnancy rate (51.1 %), which was significantly higher than that of the Score 1 and Score 2 groups. Natural pregnancy rates were 20.8 % for unilateral hydrosalpinx and 36.7 % for unilateral occlusion. Only 5 % of patients with bilateral hydrosalpinx conceived naturally. Age was a significant factor; women under 35 years had a natural pregnancy rate of 46.8 %, compared to 10.2 % in those over 40 years.

**Conclusion:**

Tubal patency status is significantly associated with pregnancy outcomes, and functional deficiencies appear to reduce fertility. The proposed HSG-based scoring system serves as a valuable predictive tool for pregnancy potential. Additionally, the presence of hydrosalpinx is associated with adverse pregnancy rates.

## Introduction

Hysterosalpingography (HSG) is a radiographic procedure used to evaluate the uterine cavity and fallopian tubes by introducing a radio-opaque substance through the cervical canal. While primarily a diagnostic modality, HSG has demonstrated therapeutic benefits, particularly with the use of oil-based contrast agents [1]. Several studies have suggested that oil-based HSG may enhance fertility [2], [3]. Despite its utility, HSG exhibits lower sensitivity and specificity compared to laparoscopy, with sensitivity and specificity estimates for tubal pathology at 0.65 and 0.83, respectively [4]. The assessment of tubal patency via HSG is particularly challenging in the presenceof periadnexal adhesions, which do not impede tubal transit [5]. Previous research indicates that bilateral tubal pathology, whether detected via HSG or laparoscopy, significantly impacts reproductive prognosis, whereas unilateral pathology has a lesser effect [6].

Traditionally, tubal patency is classified simply as occluded or patent. However, functional disorders affecting tubal transport—such as those caused by pelvic adhesions or mild endotubal pathology—also play a crucial role in fertility.^24^ To date, there is a paucity of studies comparing pregnancy rates across a spectrum of functional tubal patency statuses. This study introduces a novel classification system to evaluate the likelihood of fertility based on functional HSG findings.

## Materials and methods

This retrospective cohort study included all female patients who underwent hysterosalpingography (HSG) at our institution from January 2020 to December 2022. Inclusion criteria were as follows: (1) complete follow-up information, (2) a minimum follow-up duration of one year, and (3) completion of the HSG procedure. A total of 4461 patients underwent HSG, among which 2160 patients met the inclusion criteria and were incorporated into this study.

Inclusion criteria were defined as follows: (1) completion of the HSG procedure; (2) availability of complete follow-up data; and (3) a follow-up duration of at least one year post-procedure. All participants had a primary diagnosis of infertility and underwent HSG specifically for the assessment of tubal patency. The mean age of the cohort was 30.5 years (range: 20–45 years). Baseline reproductive history and medical background were thoroughly documented. Notably, all included patients had no prior known fertility issues at the commencement of the study.

Patients were excluded based on the following criteria:

1. Infertility attributed to male factors or secondary etiologies.

2. History of surgical or interventional procedures performed immediately prior to or following HSG.

3. Detection of uterine anomalies, such as intrauterine synechiae or polyps, during the procedure.

4. Incomplete follow-up data or loss to follow-up within the one-year period.

To minimize attrition bias, reasons for loss to follow-up (e.g., patient relocation) were meticulously recorded and analyzed. Furthermore, a comparison of baseline characteristics (age, medical history, and etiology of infertility) between included and excluded patients was conducted to assess potential selection bias; detailed statistical data are provided in the Appendix(Figure s1,Table s1). Patients were placed in the lithotomy position, and routine sterile preparation was performed. The cervix was stabilized using forceps, and 5–10 mL of contrast medium was injected via a catheter to evaluate the uterine cavity and fallopian tube patency. The procedures were conducted by a team of seven experienced radiologists. To ensure a comprehensive assessment, a delayed-phase acquisition was systematically performed 20 min post-injection to observe the distribution of the contrast medium.

Image evaluation was performed by two senior radiologists who categorized findings into: patent, unilateral or bilateral occlusion, poorly visualized tubes, or unilateral/bilateral hydrosalpinx. Functional deficiency was defined as the presence of residual contrast within the ampullary portion of the tubes on the 20-minute delayed image. This criterion was established based on prior literature suggesting that persistent contrast retention may correlate with reduced fertility, potentially indicating true pathology or transient spasm.

To ensure reproducibility, specific criteria were applied for distal occlusion and hydrosalpinx. Distal (fimbrial) occlusion was recorded when contrast opacification progressed to the ampullary region but failed to spill through the fimbrial ostium, in the absence of visually appreciable ampullary dilation. Hydrosalpinx was defined as tubular dilation of the ampullary segment with contrast retention, outlining an enlarged, tortuous ampulla; this was typically confirmed by persistent contrast within the dilated lumen on delayed-phase images. Any discrepancies in classification between the two radiologists were resolved by consensus.

A novel scoring system, detailed in Table 1, was developed for the no tubal occlusion group to predict pregnancy likelihood. The scoring system categorizes tubal patency into three Scores: Score 0 indicates patent tubes (no occlusion), Score 1 indicates unilateral functional deficiency, and Score 2 indicates bilateral functional deficiency. Tubal occlusion was categorized separately and awarded no Scores in the analysis to examine its distinct impact. Logistic regression was used to calculate odds ratios (OR) for Unilateral and bilateral functional deficiency groups, yielding ORs of 0.7265 (95 % CI: 0.5699–0.9262) and 0.4714 (95 % CI: 0.3849–0.5772), respectively. Based on actual pregnancy probabilities, scores of 1 and 2 Scores were assigned for unilateral and bilateral functional deficiencies, respectively. Data on metrosynizesis, uterine polyps, and pelvic adhesions were also collected. Patient, procedure, and outcome information were stored in a database. Follow-up phone calls assessed procedure discomfort, pregnancy status one year post-HSG, subsequent treatments, ectopic pregnancies, conception method, and live birth outcomes.

**Table 1:** A new scoring system of tube patency.

**Tubal patency**	**OR ((95 % CI)**	**Score**
Bilateraltube patent		0
Unilateral tube patent and Unilateral tube functional deficiency	0.7265(0.5699–0.9262)	1
Bilateraltube patent functional deficiency	0.4714(0.3849–0.5772)	2

patent(0 score), Unilateral tube functional deficiency（1 score）

This study was conducted in accordance with the principles outlined in the Declaration of Helsinki and adhered to all relevant ethical guidelines for research involving human participants. The research protocol was reviewed and approved by an independent ethics committee. All data were anonymized to ensure participant confidentiality and privacy.

A novel HSG-based scoring system (Table 1) was developed to predict the likelihood of pregnancy in patients without tubal occlusion. This system stratifies tubal patency into three scores: Score 0 indicates bilateral patency; Score 1 indicates unilateral functional deficiency; and Score 2 indicates bilateral functional deficiency. Cases of frank tubal occlusion were categorized separately and excluded from this scoring model to isolate the impact of functional deficiency. Logistic regression analysis was performed to validate the weighting of the scores. The analysis yielded odds ratios (OR) of 0.73 (95 % CI: 0.57–0.93) for unilateral deficiency and 0.47 (95 % CI: 0.38–0.58) for bilateral deficiency; based on these probabilities, weighted scores of 1 and 2 were assigned, respectively.

Clinical data, including the presence of intrauterine adhesions, endometrial polyps, and pelvic adhesions, were systematically recorded. All patient demographics, procedural details, and outcomes were maintained in a dedicated database. Follow-up was conducted via telephone one year post-HSG to assess pregnancy status, mode of conception (natural vs. assisted), incidence of ectopic pregnancy, and live birth outcomes. Additionally, data regarding procedural discomfort and subsequent treatments were documented.

This study was conducted in accordance with the principles of the Declaration of Helsinki. The research protocol was reviewed and approved by the institutional ethics committee. To ensure participant privacy, all data were anonymized prior to analysis.

## Statistical analyses

Inter-observer agreement between the two senior radiologists was assessed using the weighted Cohen's kappa statistic (κκ). Continuous variables were compared using One-Way ANOVA, while categorical variables, including fertility rates across groups, were analyzed using the Chi-squared test or Fisher's exact test, as appropriate. Binary logistic regression analysis was performed to estimate the odds ratios (ORs) for natural conception in the Score 1 (unilateral functional deficiency) and Score 2 (bilateral functional deficiency) groups, using Score 0 (bilateral patency) as the reference group. All statistical analyses were performed using SPSS software, version 24.0 (IBM Corp., Armonk, NY, USA). A p-value of < 0.05 was considered statistically significant.

## Results

### Study population and clinical outcomes

A total of 2461 patients were initially enrolled. After excluding 301 patients due to incomplete data, the final analysis cohort consisted of 2160 patients. Clinical outcomes following HSG are summarized in Table 2. Of the 2160 participants, 970 (44.9 %) achieved natural pregnancy within two years post-HSG; among these natural conceptions, 808 (83.2 %) resulted in live births. Additionally, 61 patients (2.8 %) conceived via artificial insemination (AI), and 775 (35.8 %) utilized in vitro fertilization (IVF). Ectopic pregnancies occurred in 39 patients (1.8 %), with a significantly higher incidence observed in the unilateral tubal occlusion group.

**Table 2:** Clinical outcomes of HSG.

**Total sample size(2160)**	
Natural pregnant (970)	44.9 %
Artificial insemination Pregnant(61)	2.8 %
IVF pregnant (775)	35.8 %
Ectopic pregnant	1.8 %
Tube patent	2.2 %
Tube deficiency	2.4 %
Tube Unilateral occlusion	4.8 %
IUAs	45/200
Endometrial polyp	16/7

IVF: In vitro fertilization IUAs: Intrauterine adhesions

### Diagnostic agreement

Regarding uterine pathology, HSG suggested intrauterine adhesions in 200 patients, of which 45 were confirmed by hysteroscopy. Conversely, while HSG identified endometrial polyps in only 7 patients, hysteroscopy confirmed the diagnosis in 16 cases.

### Pregnancy outcomes based on tubal function scores

**Fig. 1:**
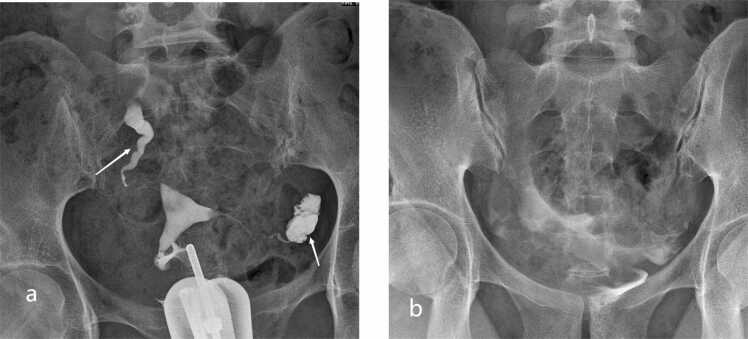
Score 0 (bilateral patent tubes) on hysterosalpingography (HSG). (a) Early/dynamic image shows contrast opacification and spill through both fallopian tube fimbrial ostia (white arrows), consistent with immediate tubal patency. (b) Delayed‑phase image (20 min) shows no retained contrast within the ampullary portions of the tubes, confirming absence of delayed contrast retention.

**Fig. 2:**
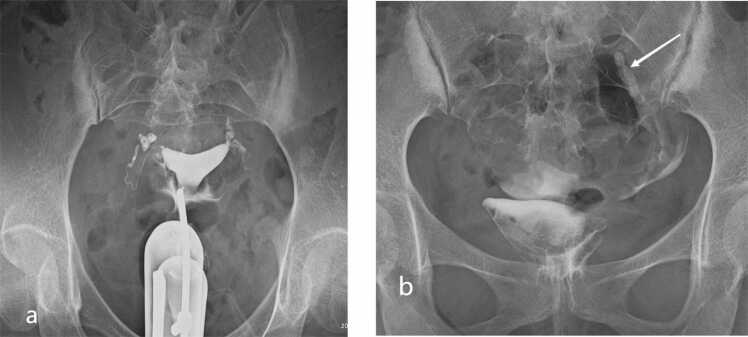
Score 1 (unilateral functional deficiency) on HSG. (a) Early/dynamic image demonstrates contrast passage to both fimbrial ostia. (b) Delayed‑phase image (20 min) shows persistent retained contrast in the left ampullary portion (white arrow), consistent with unilateral functional deficiency while the contralateral tube remains patent.

**Fig. 3:**
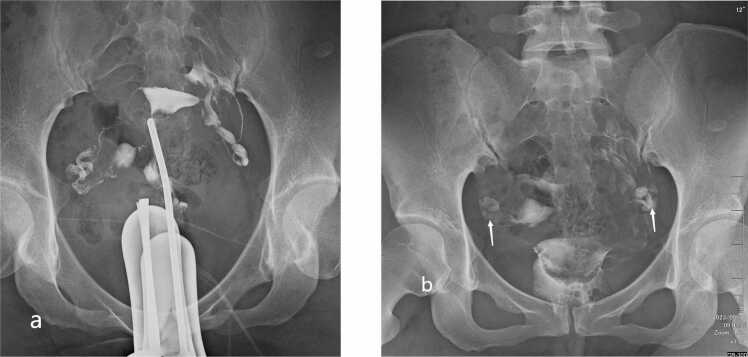
Score 2 (bilateral functional deficiency) on HSG. (a) Early/dynamic image demonstrates contrast reaching both fimbrial ostia. (b) Delayed‑phase image (20 min) shows persistent retained contrast in the ampullary portions of both fallopian tubes (white arrows), consistent with bilateral functional deficiency.

Patients were stratified based on the functional scoring system: Score 0 (bilateral patency), Score 1 (unilateral functional deficiency), and Score 2 (bilateral functional deficiency). Logistic regression analysis demonstrated that functional deficiency was significantly associated with reduced odds of natural conception. Compared to the Score 0 group, the odds ratios were 0.73 (95 % CI: 0.57–0.93, p < 0.05) for Score 1 and 0.47 (95 % CI: 0.38–0.58, p < 0.05) for Score 2 (Table 1 and Fig. 1, Fig. 2, Fig. 3).

Pregnancy and live birth rates across these groups are compared in Tables 3 and 4 and Fig. 5. The Score 0 group exhibited the highest natural pregnancy rate (51.1 %). There was a statistically significant difference in natural pregnancy rates among the three groups (p < 0.001); however, no significant difference was observed in the live birth rates among those who conceived (p = 0.072).

**Table 3:** pregnant rate and childbirth rate between different groups.

**Groups**	**Number of patients**	**Mean age**	**BMI**	**Natural pregnancy rate**	**Childbirth rate**
Unilateral occlusion (A)	150	30.5	23.31	36.70 %	65.50 %
Bilateral hydrosalpinx(B)	20	30.5	23.27	5.00 %	100 %
Unilateral hydrosalpinx(C)	24	29.7	23.35	20.80 %	80.00 %
uterine malformation(D)	18	29.8	23.30	61.10 %	81.80 %
P value	-	0.96	0.98	A vs B: p = 0.003 A vs C: p = 0.129 A vs D: p = 0.045 B vs C: p = 0.127 B vs D: p = 0.005 C vs D: p = 0.030	P＞0.1

**Table 4:** The difference of natural pregnancy rate and childbirth rate in the three groups.

Groups	Score 0	Score 1	Score 2	p
Number of patients	1019	358	671	-
Mean age	30.5	30.7	30.8	0.99
BMI	23.56	23.51	23.63	0.98
Natural pregnancy rate	51.10 %	43.30 %	33.10 %	< 0.001
Childbirth rate	82.70 %	90.30 %	84.20 %	0.072

BMI:Body mass index

### Impact of hydrosalpinx and age

**Fig. 4:**
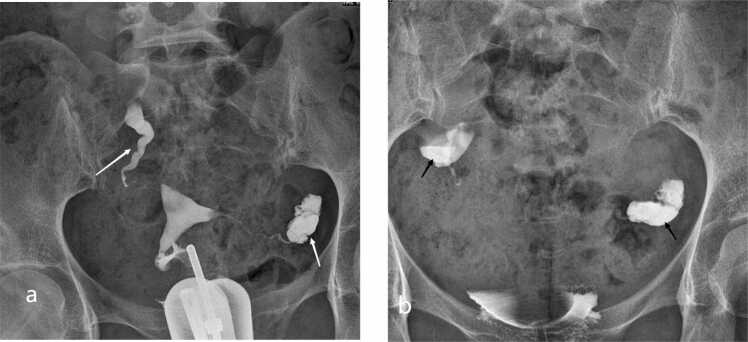
Two‑sided hydrosalpinx on HSG. (a) Initial image demonstrates failure of contrast to spill through the bilateral fimbrial ostia with marked dilatation of the ampullary segments (white arrows). (b) Delayed‑phase image (20 min) shows persistent retained contrast within the dilated ampullae (black arrows), characteristic of hydrosalpinx.

Outcomes varied significantly by tubal pathology (Fig. 4). Patients with unilateral hydrosalpinx had a notably lower natural pregnancy rate (20.8 %) compared to those with simple unilateral occlusion (36.7 %). In cases of bilateral hydrosalpinx, the natural pregnancy rate dropped to only 5 %. Despite these differences in conception rates, the live birth rate remained consistent across all categories.

**Fig. 5:**
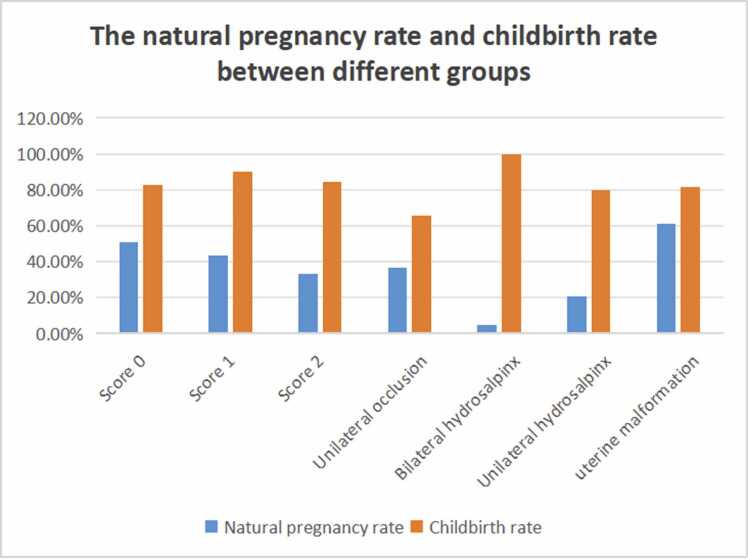
Natural pregnancy and live‑birth rates by tubal patency category. Bar chart illustrates the proportion of patients achieving natural conception and subsequent live birth within two years after HSG for each diagnostic group (Score 0 = bilateral patent; Score 1 = unilateral functional deficiency; Score 2 = bilateral functional deficiency; additional bars denote one‑sided and two‑sided hydrosalpinx and complete occlusion). Error bars indicate 95 % confidence intervals.

Age-stratified analysis (Table 5) revealed a significant inverse relationship between age and fertility. Women under 35 years demonstrated a significantly higher natural pregnancy rate (46.8 %) compared to older cohorts, while the lowest rate was observed in patients over 40 years (10.2 %).

**Table 5:** pregnancy rate between different age groups.

**Age**	**Natural pregnant rate**	**P value**
Below 35 years	46.8 %	＜0.001
35–40 years	33.2 %	
Over 40 years	10.2 %	

## Discussion

Previous studies have consistently demonstrated that oil-based HSG contrast media may enhance pregnancy outcomes compared to water-based media [3], [7]. However, the prognostic utility of HSG remains a subject of debate, primarily due to its limited ability to predict spontaneous conception [8]. Traditional classification systems typically categorize tubes simply as "patent" or "occluded," often overlooking the nuances of functional tubal disease and concurrent uterine pathologies that significantly impact fertility.

In 1983, Norderskjold and Ahlgren reported on 433 subfertile women who underwent laparoscopy, finding that adhesions had a similar impact on reproductive outcomes as unilateral tubal occlusion, with relative risks of 0.74 (95 % CI 0.57–0.98) and 0.73 (CI 0.39–1.4), respectively [9]. This study introduced several subgroups to compare pregnancy rates and predict outcomes based on different types of tubal patency, aiding in treatment decisions. Compared to laparoscopy, HSG generally shows reduced sensitivity for detecting tubal occlusion [4], [6], indicating its limited accuracy in diagnosing such conditions. In this study, we calculated pregnancy rates without diagnosing occlusion for poorly visible tubes on HSG.

The study included 2160 patients, with a natural pregnancy rate of 44.9 % (970/2160), aligning with previous findings [3], [10]. Within subgroups, the absolute patent (Score 0) group had the highest natural pregnancy and childbirth rates (51.1 %), while the Score 2 group had only a 33.1 % pregnancy rate, suggesting that functional deficiencies may reduce pregnancy rates. Interestingly, tubes with Unilateral occlusion or tubal excision showed higher pregnancy rates than the Score 2 group, This finding suggests a critical clinical insight: tubal quality may be more determinative of fertility than mere tubal quantity. Only 5 % of patients with bilateral hydrosalpinx achieved pregnancy, consistent with previous research indicating that hydrosalpinx can decrease pregnancy rates through mechanisms like mechanical washout and toxic effects on embryos [11], [12]. Further prospective studies are needed to determine if therapeutic salpingectomy or tubal blockage can improve IVF or spontaneous pregnancy rates in women with hydrosalpinx [13], [14].

Age was a significant factor in pregnancy rates, with those under 35 years showing a rate of 46.8 %, while those over 40 had a rate of only 10.2 %. This aligns with findings that fertility declines rapidly after age 40 [15], [16]. The study found a 35.8 % pregnancy rate with IVF, higher than previous studies, possibly reflecting IVF's acceptance in China's first-tier cities. However, artificial insemination resulted in only 2.8 % of pregnancies [3]. Ectopic pregnancies occurred in 1.8 % of patients, consistent with earlier studies [17], [18], increased from 1.4 % at age 21–6.9 % at age 44[19] which also identified age over 35 as a significant risk factor. Our study found a 4.1 % ectopic pregnancy rate in women over 35, consistent with other research, and a higher rate (4.8 %) in patients with Unilateral occlusion, warranting further investigation.

HSG initially detected 200 cases of intrauterine adhesions (IUAs), but only 45 were confirmed by laparoscopy. HSG showed a sensitivity of 75–81 %, specificity of 80 %, and positive predictive value of 50 % when compared to hysteroscopy for diagnosing IUAs [20]. Endometrial polyps were confirmed in 16 patients via hysteroscopy, while HSG identified 7 cases. Hysterosalpingography, while having higher sensitivity (98 %), showed lower specificity (34.6 %) compared to hysteroscopy for diagnosing endometrial polyps [21].

## Limitations

This study has several limitations. First, its retrospective design relies on the accuracy of historical record-keeping, which may introduce information bias. Second, a subset of patients was lost to follow-up or provided unclear information, potentially influencing outcome calculations. Third, as a single-center study, variations in HSG technique compared to other institutions may limit generalizability. Fourth, while HSG is a primary screening tool, its diagnostic accuracy for subtle intrauterine pathologies is inferior to hysteroscopy, which may have affected the classification of some patients. Finally, the proposed scoring system was internally derived; external validation in independent cohorts is essential before broad clinical application.

## Conclusion

This study demonstrates that pregnancy rates are significantly associated not only with tubal patency but also with functional tubal quality. The proposed novel scoring system offers a more nuanced prediction of future pregnancy than traditional binary classifications. Specifically, bilateral functional deficiency appears to carry a worse prognosis than simple unilateral occlusion. Furthermore, the presence of hydrosalpinx remains a profound negative predictor for natural conception. These findings suggest that evaluating tubal function, alongside patency, is crucial for counseling subfertile couples.

## CRediT authorship contribution statement

**Huijun Yang:** Writing – original draft. **Saiming Cheng:** Investigation, Funding acquisition. **Yali Xu:** Writing – original draft, Methodology. **Jiejun Cheng:** Writing – review & editing. **Haixia Zhang:** Resources, Data curation. **Feng Gao:** Supervision, Conceptualization.

## Ethics approval and consent to participate

This study was approved by the Ethics Committee of the Shanghai First Maternity and Infant Hospital (registration number: KS22281) and was conducted by FengGao. Written patient informed consent for the data used in our study was waived because of the retrospective nature of our study. The study was performed following the 1964 Helsinki Declaration and its later amendments or comparable ethical standards. The study complies with all regulations. All methods were performed under the relevant guidelines and regulations. Written informed consent s was obtained from all patients before examination.

## Consent for publication

Not applicable

## Funding

This study was supported by grants from the Shang First Maternity and Infant Hospital Talent Project (2022RC04) and the Shanghai Tansuozhe Project (23TS1401000).

## Declaration of Competing Interest

The authors declare that they have no competing interests.

## Data Availability

The datasets generated and/or analyzed during the current study are not publicly available because they contain the patients’ personal information, but are available from the corresponding author on reasonable request.

## Appendix A Supplementary material

Supplementary material

Patient selected Flowchart

## References

[1] Schwabe M.G., Shapiro S.S., Haning R.V. (1983). Hysterosalpingography with oil contrast medium enhances fertility in patients with infertility of unknown etiology. Fertil Steril.

[2] van Welie N., Rosielle K., Dreyer K., van Rijswijk J., Lambalk C.B., van Geloven N., Mijatovic V., Mol B.W.J., van Eekelen R. (2020). Group HOS: How long does the fertility-enhancing effect of hysterosalpingography with oil-based contrast last?. Reprod Biomed Online.

[3] Dreyer K., van Rijswijk J., Mijatovic V., Goddijn M., Verhoeve H.R., van Rooij I.A.J., Hoek A., Bourdrez P., Nap A.W., Rijnsaardt-Lukassen H.G.M. (2017). Oil-Based or Water-Based Contrast for Hysterosalpingography in Infertile Women. N Engl J Med.

[4] Swart P., Mol B.W., van der Veen F., van Beurden M., Redekop W.K., Bossuyt P.M. (1995). The accuracy of hysterosalpingography in the diagnosis of tubal pathology: a meta-analysis. Fertil Steril.

[5] Swolin K., Rosencrantz M. (1972). Laparoscopy vs. hysterosalpingography in sterility investigations. A comparative study. Fertil Steril.

[6] Mol B.W., Collins J.A., Burrows E.A., van der Veen F., Bossuyt P.M. (1999). Comparison of hysterosalpingography and laparoscopy in predicting fertility outcome. Hum Reprod.

[7] Fang F., Bai Y., Zhang Y., Faramand A. (2018). Oil-based versus water-based contrast for hysterosalpingography in infertile women: a systematic review and meta-analysis of randomized controlled trials. Fertil Steril.

[8] Maas J.W., Evers J.L., ter, Riet G., Kessels A.G. (1997). Pregnancy rate following normal versus abnormal hysterosalpingography findings: a meta-analysis. Gynecol Obstet Invest.

[9] Nordenskjold F., Ahlgren M. (1983). Laparoscopy in female infertility. Diagnosis and prognosis for subsequent pregnancy. Acta Obstet Gynecol Scand.

[10] Li H., Ren Y., Yan J., Huang M., Zheng B., Luo X., Huang S., Cai S. (2022). Fertility Outcome and Safety of Ethiodized Poppy Seed Oil for Hysterosalpingography in 1,053 Infertile Patients: A Real-World Study. Front Med (Lausanne).

[11] Yang X., Zhu L., Le F., Lou H., Zhao W., Pan P., Zou Y., Jin F. (2020). Proximal Fallopian Tubal Embolization by Interventional Radiology prior to Embryo Transfer in Infertile Patients with Hydrosalpinx: A Prospective Study of an Off-label Treatment. J Minim Invasive Gynecol.

[12] Bashiri A., Halper K.I., Orvieto R. (2018). Recurrent Implantation Failure-update overview on etiology, diagnosis, treatment and future directions. Reprod Biol Endocrinol.

[13] Yip J.Y., Kanneganti A., Binte Ahmad N., Lim M.X.K., Chew S.L.S., Huang Z. (2023). Optimizing intrauterine insemination and spontaneous conception in women with unilateral hydrosalpinx or tubal pathology: A systematic review and narrative synthesis. Eur J Obstet Gynecol Reprod Biol.

[14] Broeze K.A., Opmeer B.C., Van Geloven N., Coppus S.F., Collins J.A., Den Hartog J.E., Van der Linden P.J., Marianowski P., Ng E.H., Van der Steeg J.W. (2011). Are patient characteristics associated with the accuracy of hysterosalpingography in diagnosing tubal pathology? An individual patient data meta-analysis. Hum Reprod Update.

[15] Dunson D.B., Colombo B., Baird D.D. (2002). Changes with age in the level and duration of fertility in the menstrual cycle. Hum Reprod.

[16] Deatsman S., Vasilopoulos T., Rhoton-Vlasak A. (2016). Age and Fertility: A Study on Patient Awareness. JBRA Assist Reprod.

[17] Nybo Andersen A.M., Wohlfahrt J., Christens P., Olsen J., Melbye M. (2000). Maternal age and fetal loss: population based register linkage study. BMJ.

[18] Goddijn M., van der Veen F., Schuring-Blom G.H., Ankum W.M., Leschot N.J. (1996). Cytogenetic characteristics of ectopic pregnancy. Hum Reprod.

[19] Farquhar C.M. (2005). Ectopic pregnancy. Lancet.

[20] Amin T.N., Saridogan E., Jurkovic D. (2015). Ultrasound and intrauterine adhesions: a novel structured approach to diagnosis and management. Ultrasound Obstet Gynecol.

[21] Preutthipan S., Linasmita V. (2003). A prospective comparative study between hysterosalpingography and hysteroscopy in the detection of intrauterine pathology in patients with infertility. J Obstet Gynaecol Res.

