# Supplementary material for: Evaluation of pregnancy outcomes using a novel hysterosalpingography scoring system for tubal patency
Source: Eur J Obstet Gynecol Reprod Biol X. 2025 Nov 27;29:100437. doi: 10.1016/j.eurox.2025.100437 (PMC12721197; doi:10.1016/j.eurox.2025.100437)
Supplement: Supplementary file 1 — Supplementary material [file mmc1.docx]

Figure s1. Patient selected Flowchart
