# Supplementary figures and images for: Evaluation of pregnancy outcomes using a novel hysterosalpingography scoring system for tubal patency
Source: Eur J Obstet Gynecol Reprod Biol X. 2025 Nov 27;29:100437. doi: 10.1016/j.eurox.2025.100437 (PMC12721197; doi:10.1016/j.eurox.2025.100437)

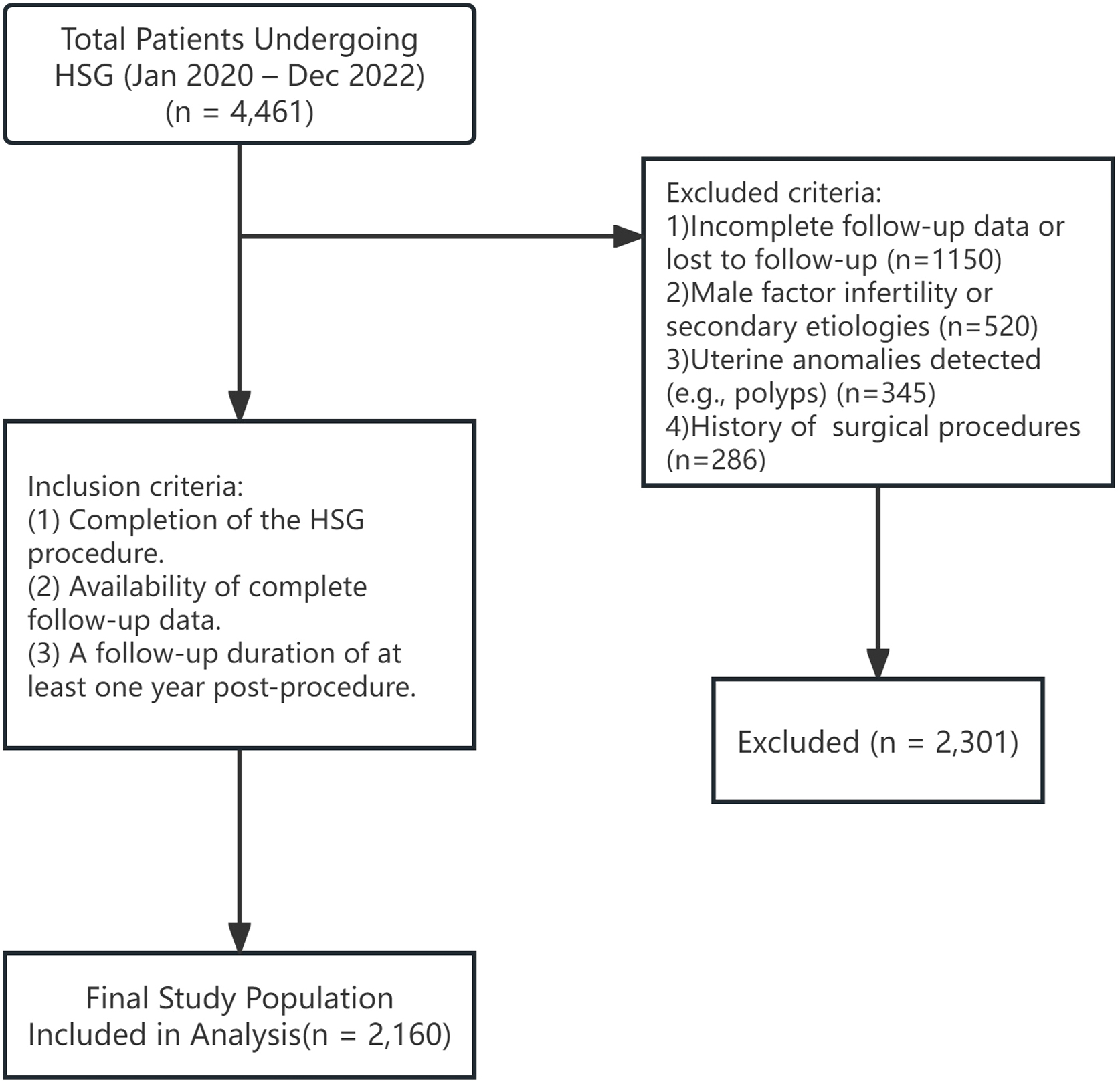

Supplement: Supplementary file 2 — Patient selected Flowchart [file mmc2.jpg]
